# A Novel Approach on Microwave Hyperthermia

**DOI:** 10.3390/diagnostics11030493

**Published:** 2021-03-10

**Authors:** Gulsah Altintas, Ibrahim Akduman, Aleksandar Janjic, Tuba Yilmaz

**Affiliations:** 1 Department of Electronics and Communication Engineering, İstanbul Technical University, 34469 İstanbul, Turkey; akduman@itu.edu.tr (I.A.); janjic19@itu.edu.tr (A.J.); tuba.yilmaz@itu.edu.tr (T.Y.); 2 Mitos Medical Technologies, 34469 İstanbul, Turkey

**Keywords:** microwave hyperthermia, focusing, breast hyperthermia

## Abstract

Microwave hyperthermia (MH) requires the selective focusing of microwave energy on the targeted region while minimally affecting the healthy tissue. Emerging from the simple nature of the linear antenna arrays, this work demonstrates focusing maps as an application guide for MH focusing by adjusting the antenna phase values. The focusing of the heating potential (HP) on different density breast models is performed via the proposed method using Vivaldi antennas. The effect of the tumor conductivity on the focusing is discussed. As a straightforward approach and utilizing the Vivaldi antennas, the system can be further combined with MH monitoring application.

## 1. Introduction

Breast cancer is the most frequent cancer type amongst women all over the world, constituting almost 30% of the cancer malignancies [1]. The most popular therapy techniques for breast cancer are radiotherapy, chemotherapy and the surgical procedures. Similar to other therapeutic techniques, chemotherapy has its own drawbacks, such that it does not only destroys the rapid growing cancer cells but also destroys healthy rapid growing cells, such as the hair, mouth and gastro-intestinal cells [1]. The longer the treatment, the more serious the side effects become. Furthermore, the economic burden of the treatment also increases with each session.

An alternative approach is to combine breast cancer treatment modalities to increase the effectiveness of the treatment. It is known that elevated temperature levels at the target region attract the temperature-sensitive drugs used in chemotherapy and increase the effectiveness of the drugs (or anti-cancer agents) on the heated area instead of the healthy region [2]. This approach enables effective and shorter chemotherapy sessions with targeted drug delivery while decreasing the toxicity in healthy tissues due to chemotherapy. Increasing the temperature in body tissue from 40 to 43 °C is the main purpose of hyperthermia studies. Further heat delivery to the tumor denatures the nucleoic, cytoplasmic or membrane proteins, causing a cytotoxic effect on the malignant cells; increasing the tissue temperature over a threshold, generally 43 °C, is defined as thermal ablation, which can be used as a solo treatment technique. Given the fact that hyperthermia is a destructive operation on the targeted cells, accurate focusing of the microwave energy to the target region is essential. One of the critical aspects of hyperthermia is to increase the temperature of the targeted area while restraining it such that the non-targeted/healthy regions remain under the denaturation limits.

Electromagnetic (EM) fields in 434, 915 or 2450 MHz industrial, scientific and medical (ISM) frequency bands are the most preferred frequencies when performing microwave hyperthermia (MH). Microwave frequencies are non-ionizing, and their application is non-invasive, such that MH does not directly harm the cells, so targeting becomes the main concern. MH is only destructive when the applied EM waves are in-phase at the target and the deposited energy increases the temperature over 43 °C. Simultaneous radiation with different amplitude and the phase from numerous locations is an accepted approach to achieve effective targeting of the tumor. Tissue medium is lossy, the EM wave loses power as it travels through the body, and the blood stream maintains the temperature homeostasis at the remaining tissues, making MH a safe treatment mechanism for the whole body.

Numerous studies have been conducted for breast microwave hyperthermia. In general, the breast is placed in a cavity encircled by a number of antennas; next, the amplitude and the phase are optimized in a patient-specific manner for each antenna. Two-dimensional specific absorption rate (SAR) focusing algorithms have been developed by Iero et al. [3,4], which is then extended to 3D and named as optimal constrained power focusing (FOCO) [5]. In previously reported works, simple or semi-realistic phantoms have been used. Bellizzi et al. [6] used a multi-frequency 2D FOCO approach for the focusing; the effectiveness of the developed algorithm was evaluated for different breast densities. Elkayal et al. [7], Curto et al. [8] and Merunka et al. [9] also used simple phantoms but analyzed the focusing of the temperature instead of SAR. A large number of reported studies have adapted circular antenna array configuration or multi-layered cylindrically arranged antennas.

Microwave hyperthermia has also been performed with linear antenna arrays in the literature. Fenn [10] is among the pioneers in this subject; he used a microwave waveguide for radiation and compressed the breast for better data acquisition as well as a stable procedure. In this work, an electric diode was used to monitor the temperature, which is an invasive method and not feasible to human breast. Dooley et al. [11] continued Fenn’s work and reported a clinical study, in which local anesthesia is applied to the breast and punctured so that a sensor catheter is inserted into the nick; where the sensor monitors the electric field and the temperature. Nguyen et al. [12] positioned 4 sub-arrays on the 4 sides of the breast; each sub-array was in linear fashion. Three-dimensional realistic breast models were used, and both heating potential and temperature changes were presented. The utilized method aims to solve the optimization problem in a trust-region framework while trying to maximize the ratio of power dissipated on the tumor to power dissipated on the healthy tissue. In [13], they extend their work on different tumor sizes and locations, but not different breast sizes and densities. The same year, an experiment study was introduced [14], in which the Particle Swarm Optimization algorithm was applied. In addition to breast cancer treatment applications, MH is used for the treatment of other diseases, including, but not limited to, malignant tumors in the brain, bones, neck, bladder and benign tumors in prostate tissues. In [15], a beamforming method was proposed for non-invasive MH of pediatric brain cancer. The survival of bone cancer cell lines induced with gold nanoparticles was evaluated in [16] to investigate the effectiveness of combined MH and chemotherapy treatment. Clinical trials of a slot antenna were presented in [17] for invasive localized MH application on a neck tumor. The efficacy of combined MH and chemotherapy for bladder cancer treatment was investigated in [18]. In [19], localized hyperthermia for the treatment of benign prostatic hyperplasia was given. Lastly, MH was also proposed to be utilized for boosting muscle recovery after injuries [20].

The goal of this work is to provide a simple framework that can be used for focusing EM energy to tumors located in breast medium without adopting complex optimization schemes. We propose a linearly distributed antenna configuration for breast MH. Vivaldi antennas are adapted and simulated using multiphysics simulation software COMSOL Multiphysics at the 915 MHz frequency band. First, heating potential (HP) in a linearly changing phantom is considered, and “focusing maps” (FMs) are formed as a guide. The realistic phantoms, which are obtained from magnetic resonance images, are simulated according to the guidance of the FMs. 

The remainder of this paper is organized as follows: In Methods, the proposed strategy to focus the heating potential to target region is discussed. The Results section analyzes proof of concept examples and stresses the importance of tumor conductivity. The obtained results and the performance of the proposed method are reviewed in the Discussion.

## 2. Materials and Methods 

### 2.1. Heating Potential 

Penne’s bio-heat equation (Equation (1)) [21] governs the heat transfer phenomena within the biological systems:

(1) $$C_{p}\rho \frac{\partial T}{\partial t}=\nabla \cdot \left(K\nabla T\right)+A_{0}+Q_{0}-B\left(T-T_{b}\right)$$

where $C_{p}$ is specific heat capacity, ρ is the density, K is the thermal conductivity, T is the temperature,$\;T_{b}$ is the blood temperature, $A_{0}$ is the metabolic heat generation, B is the capillary blood perfusion coefficient and they are all tissue-specific terms; whereas $Q_{0}$ is the heating potential (HP) and depends on the square of the electric field, $Q_{0}=0.5\sigma {\left|E\right|}^{2}\;W/m^{3}$, where E is the electric field and σ (S/m) is the electrical conductivity. Iero et al. [4] showed, via Green’s function approach, that the maxima of HP and the temperature are located at the same position, assuming K and B are constants and in a steady state. In this paper, we use the HP as a criterion to assess our study.

### 2.2. Antenna Configuration 

**Figure 1:**
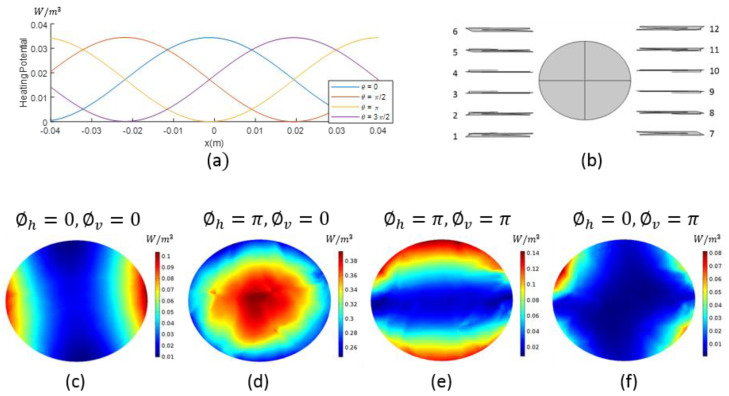
(**a**) Superposition of two opposite propagating plane electromagnetic waves, where θ is the phase difference of the waves; (**b**) Top view of the simulation configuration with antenna numbering and homogeneous fat phantom; (**c**–**f**) Heating potential of homogeneous fat phantom with the given sub-antenna arrays phase adjustments.

We conducted simulations in COMSOL Multiphysics (COMSOL AB, Stockholm, Sweden) using the frequency domain study in radio frequency module. Furthermore, 12 Vivaldi antennas were used in the simulations. The Vivaldi antenna is a wide band antenna, and originally, it is used for microwave imaging studies. 6 antennas were placed linearly 2 cm apart from each other and 0.5 cm away from the left-most corner of the breast geometry, and other 6 antennas were placed in a same manner to the right side of the breast geometry, the general geometry is given in Figure 1b. Antennas were fed with a uniform lumped port, and the amplitude of the antennas was 1 volt and kept constant throughout the study. The phases of the antennas were adjusted as the method requires. 

### 2.3. Focusing

Figure 1a shows the HP in a 1-dimensional medium corresponding to the superposition of two plane EM waves propagating with 915 MHz in opposite directions, where θ denotes the phase difference between the two EM waves. Homogenous medium used here is equivalent to fat tissue with 0.0092 S/m conductivity (σ) and 3.9459 units relative permittivity ($\epsilon_{r}$) [22]. The peak value of HP is shifted through the medium with changing θ. Starting from this point, we applied the same reasoning to 3-dimensional system. Figure 1b displays the top-view of a 3-dimensional homogeneous fat phantom, which is an ellipsoid with 4.5 × 3.85 × 7.25 cm semi-axis dimensions and the linearly placed Vivaldi antennas. The ellipsoid is cut to half to simulate the breast shape. We used 6 Vivaldi antennas at both the left and right sides of the phantom, where the phase difference between the sub-arrays were changed. We used 2 sub-arrays: sub-array-1 consists of antennas 7, 8, 9, 10, 11 and 12, and sub-array-2 consists of antennas 1, 2, 3, 7, 8 and 9 (Figure 1b). These sub-arrays divide the antenna system into vertical and horizontal sections. 

Starting with the same phase and the amplitude for the antennas, we changed the phase of the sub-array-1 to $\emptyset_{h}$ denoting the horizontal phase difference. Figure 1c–f illustrate the HP of the z = 0 slice of the 3-dimensional homogenous fat phantom simulation. Figure 1c,d display the HP where $\emptyset_{h}$ is 0 and π, respectively. With this phase change, we were able to move the focus from horizontal sides to the center of the homogeneous phantom; $\emptyset_{h}$ can be used to selectively focus on the horizontal axis. Adding vertical phase difference $\emptyset_{v}$ to sub-array-2, we obtained the following phase values for the antennas:$\;\emptyset_{1},\emptyset_{2},\emptyset_{3}=\emptyset_{v}$;$\;\emptyset_{4},\emptyset_{5},\emptyset_{6}=0$;$\;\emptyset_{7},\emptyset_{8},\emptyset_{9}=\emptyset_{h}+\emptyset_{v}$;$\;\emptyset_{10},\emptyset_{11},\emptyset_{12}=\emptyset_{h}$. Figure 1d,e display the HP where $\emptyset_{v}$ is 0 and π, respectively, and the focus is shifted from the center to the vertical edges, providing the opportunity for selective focusing on the vertical axis. The HP in Figure 1f is focused in a diagonal fashion. From the presented analysis, we can conclude that it is possible to move the focus of HP as desired by just appropriately changing $\emptyset_{h\;}\mathrm{and}\;\emptyset_{v}$ phase values. 

**Figure 2:**
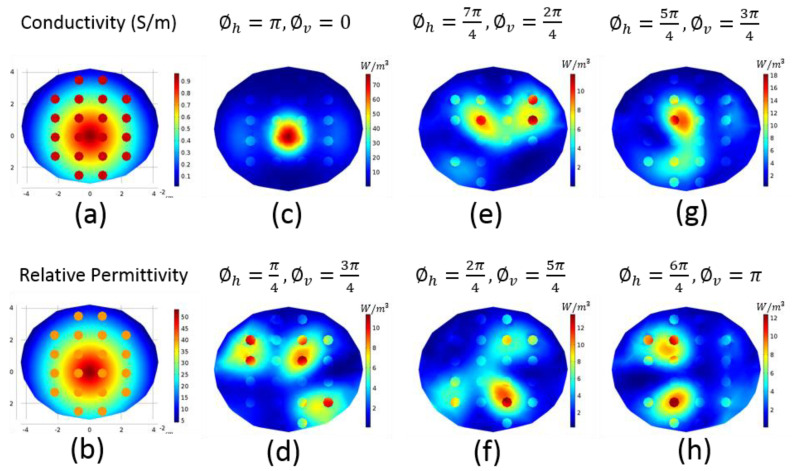
(**a**) Electrical conductivity and (**b**) relative permittivity of the fading breast model; (**c**–**h**) Focusing maps (heating potential of the fading model where the phases of the sub-antenna arrays are adjusted as given above the individual figures).

The wave behavior changes according to the medium. In general, breast has density (glandular tissue) at the center and fat tissue located closer to the surface. Considering this fact, a fading breast model, the geometry given in Figure 1b, was prepared to mimic the breast tissue in the most generic way. The center of the fading model was set to the electrical properties of glandular tissue, and the edges were set to the electrical properties of fat tissue; electrical properties were then changed linearly from the center to edges forming the fading model. Spherical incidences with 0.3 cm radius, $\epsilon_{r}$ = 40 and σ = 0.9 S/m were placed to represent possible tumors with different locations. These incidences were placed at *z* = −2 cm slice, in every 1.5 cm in x-direction and in every 1.2 cm in y-direction with a total of 20 incidences. The electrical properties of the fading model are given in Figure 2a,b. The ($\emptyset_{h},\emptyset_{v}$) phase pair was changed to the integer multiples of $\frac{\pi }{4}$, ranging between [0,$\frac{7\pi }{4}$], and the HP of this model was calculated with each phase pair. These HP maps are called “focusing maps” (FMs) for further reference. Figure 2c–h display a few FMs with different ($\emptyset_{h},\emptyset_{v}$) pairs. The FM provides a set of focused models, from which the user can choose the optimum phase pair for the desired target.

### 2.4. Realistic Numeric Phantoms 

Dielectric properties for the realistic numeric phantoms used in this study were taken from the phantom repository of University of Wisconsin Cross-Disciplinary Electromagnetics Laboratory [22,23]. Two breast models were used, with ID 062204 and ID 012304, categorized as heterogeneously dense (breast model-I) and very dense breast (breast model-II), respectively. Both in diagnostics and therapy, tumors embedded inside the dense tissue are complicated to operate with, and studying dense breast models approves the validity of the method. Relative permittivity and electrical conductivity values were calculated in MATLAB (The MathWorks, Natick, MA) for each 0.5 × 0.5 × 0.5 mm cubic voxel using the provided Debye parameters for the corresponding breast phantom. These breast models were then transferred to COMSOL Multiphysics. 

**Figure 3:**
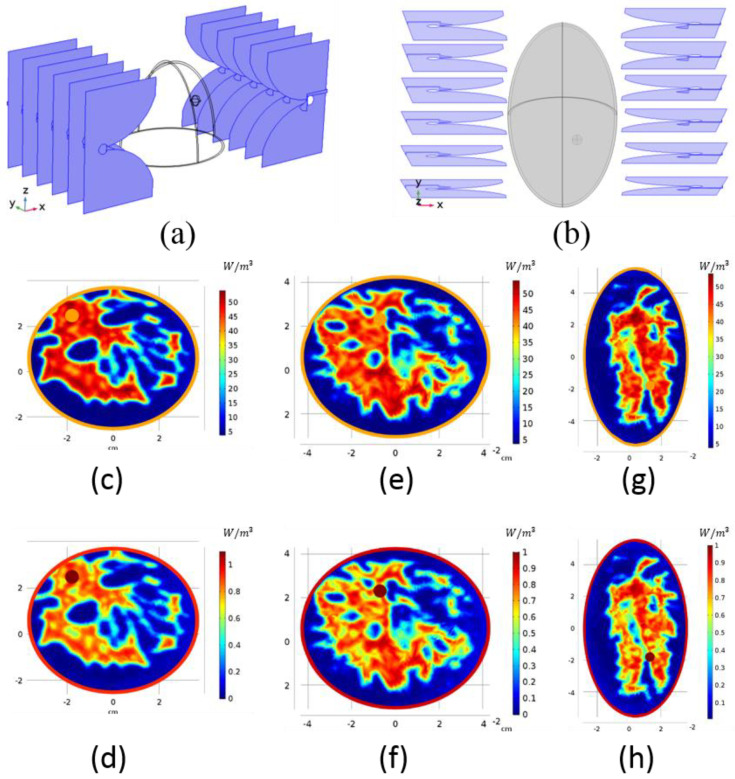
Simulation configuration in COMSOL environment with Vivaldi antennas and semi-ellipsoid geometries used as: (**a**) breast model-I; (**b**) breast model-II. Electrical properties of the ellipsoid shaped breast models: (**c**) relative permittivity of breast model-I at *z* = −2 cm; (**d**) electrical conductivity of breast model-I at *z* = −2 cm; (**e**) relative permittivity of breast model-I at *z* = 0 cm; (**f**) electrical conductivity of breast model-I at *z* = 0 cm; (**g**) relative permittivity of breast model-II at *z* = −2 cm; (**h**) electrical conductivity of breast model-II at *z* = −2 cm.

Due to the asymmetric and complicated shape of the breasts, COMSOL Multiphysics failed to converge during the simulations. Therefore, simpler shapes were formed inside the program using its geometry interface. An ellipsoid with 4.5 × 3.85 × 7.25 cm semi-axis dimensions fitted well to the shape of breast model-I (Figure 3a), and an ellipsoid with 3.4 × 5.8 × 7.25 cm semi-axis dimensions was used for breast model-II (Figure 3b). A skin layer of 0.15 cm was added to the ellipsoid, consistent with the original data. Dielectric properties of the skin were also calculated from the Debye parameters. The ellipsoid was cut such that the height of the breast remained as 6.75 cm. Although the shapes of the breasts were not original, we mapped the dielectric values formed from the corresponding Debye parameters to the simpler breast phantoms. There were no cancerous tissues in the repository; therefore, a sphere with a 0.3 cm radius was formed as the tumor tissue. Dielectric properties of the breasts at 915 MHz at *z* = −2 cm and *z* = 0 cm slices are given in Figure 3c–h. Relative permittivity of the tumor was kept constant, $\epsilon_{r}=40$ units, throughout this study, but the conductivity of the tumor was changed.

## 3. Results

**Figure 4:**
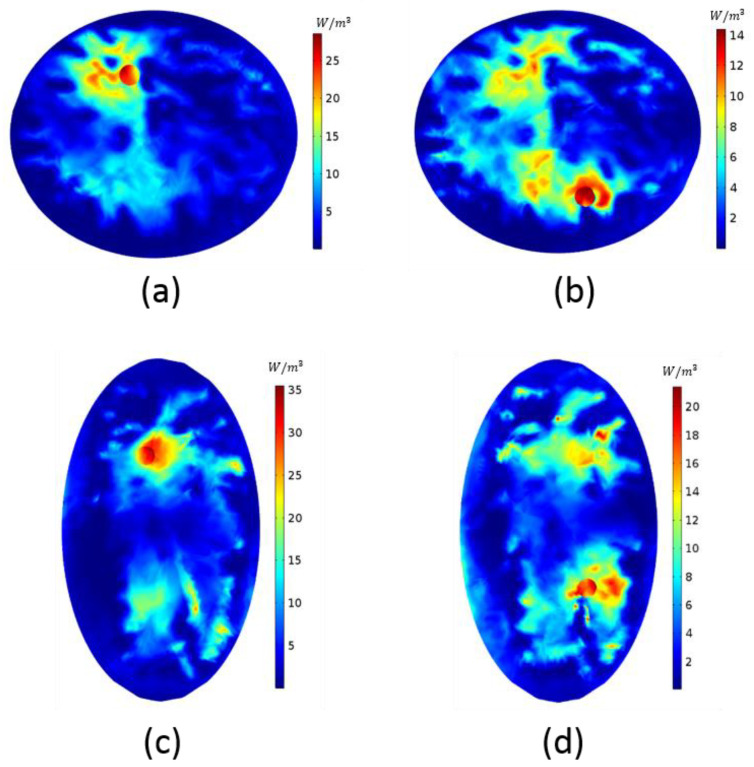
Heating potential of (**a**,**b**) breast model-I with different tumor locations; (**c**,**d**) breast model-II with different tumor locations.

A spherical tumor with 1 S/m conductivity was placed into *z* = −2 cm slice of breast model-I into the location shown in Figure 4a. In Figure 2g, the FM with the closest focus is shown; for this tumor location, the optimal phase pair is $\;\emptyset_{h}=5\pi /4,\emptyset_{v}=3\pi /4$. Applying this pair to breast model-I, we obtained the HP map in Figure 4a. Figure 4b displays the HP of another tumor location. The FM in Figure 2f obtained by $\emptyset_{h}=2\pi /4,\emptyset_{v}=5\pi /4$ is consistent with the desired focus location; therefore, this pair of phase values was applied to the breast model. Focus obtained by the FM agrees with the focus acquired in Figure 4b. 

Figure 4c,d illustrates the focusing on breast model-II. A spherical tumor with 1 S/m conductivity was placed at different locations on *z* = −2 cm slice. Although the shapes of the acquired FM and this breast model were not similar, it was expected that the trend would not differ, and we used the same FM to determine the optimal phase pair. The tumor location of Figure 4c requires a focusing similar to the FM in Figure 2h with $\emptyset_{h}=6\pi /4,\emptyset_{v}=\pi$ phase values. The tumor location in Figure 4d is similar to the tumor location in Figure 4b; when applying the same phase pair, the HP of the tumor was focused expectedly. Even though we used the same phase pairs for Figure 4b,d, the HPs were not the same. The shape of the breasts and the electrical properties were different from each other; therefore, one cannot anticipate the exact same result. However, the focusing occurred as intended. 

**Figure 5:**
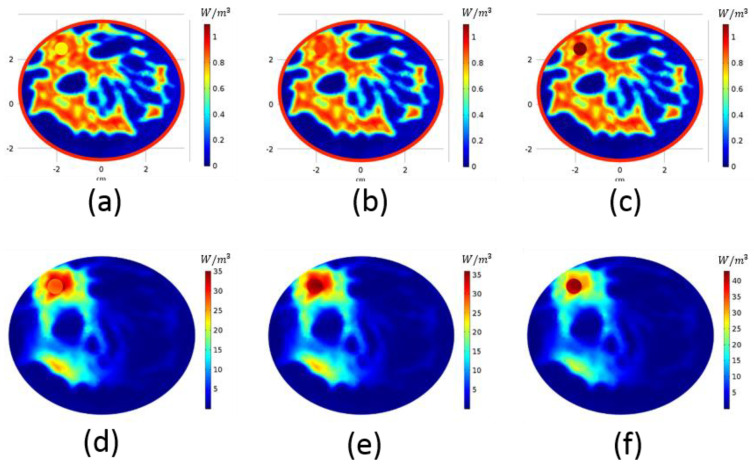
Electrical conductivity map of breast model-I with tumor of (**a**) 0.7 S/m, (**b**) 0.9 S/m and (**c**) 1.1 S/m. Heating potential distribution of breast model-I with tumor of (**d**) 0.7 S/m, (**e**) 0.9 S/m and (**f**) 1.1 S/m.

HP is directly proportional to the conductivity. We conducted a study with a z = 0 slice of breast model-I, the electrical conductivities of which are given in Figure 5a–c, to observe the effect of tumor conductivity on the focusing. Three different tumor conductivity values, 0.7 S/m, 0.9 S/m and 1.1 S/m, were used. Figure 5 gives the conductivity maps and the corresponding HP distributions, where the $\emptyset_{h}=6\pi /4,\emptyset_{v}=\pi$ phase pair (Figure 2h) was used for focusing. When the tumor conductivity is higher, the HP is higher on the tumor and restricted to the tumor geometry; however, when the conductivity value is comparable to or a little lower than the surrounding tissue, the HP focus is no longer confined in the tumor but also to the neighboring tissue. Focus can be steered to a certain region via phase configuration, despite low conductivity values. 

## 4. Discussion

In this article, a study on focusing of the HP with linearly distributed Vivaldi antennas in order to perform microwave breast hyperthermia is presented. A “Focusing Map” is introduced as a guide for phase configuration to achieve the desired focus on the breast: it overcomes the trial–error method of the values as in [9]. Since every breast is different in shape and density, a generic fading model was used for the FM, and its scope was validated on both breast model-I and breast model-II obtained from real MRI data, which have different shapes and densities.

Among other ISM frequencies, the best performance was obtained at 915 MHz. We were able to transfer heat to a deep-seated tumor with a 0.3 cm radius, which corresponds to an early-stage tumor. With larger tumors, focusing is even more evident. It is harder to image and operate on the tumors buried inside the dense tissue; the effectiveness of this method is demonstrated by embedding the tumors into the glandular tissue. We show that focusing can be achieved with different tumor conductivities, whereas, as the conductivity of the tumor increases, hot spots at the neighboring tissue decrease. It is important to mention that even though the tumor is less conductive than the surrounding region, with the proper calibration of the antenna phase, HP can be focused on the tumor. 

FMs are easy to comprehend and apply during the treatment as opposed to complicated focusing algorithms [3,4,5,6,7,8,9]. Those algorithms need to be run before the treatment, and the user has no control over the program, and since they are patient-specific, the patient also needs to wait for the algorithm to converge. Note that the convergence is not guaranteed. With circular array of antennas, the user has no other choice but utilizing a focusing algorithm, since the behavior of the created field is unimaginable by the end-user. In contrast, linearly distributed antennas are practical to adjust with a pair of trained eyes. Working only with the phase of the antennas brings extra ease to the application. Alternatively, the range of the utilized phase values can be increased by a smaller step size. This would enable more precise focusing, but it may counteract the manageable nature of the proposed method.

Lastly, when the Penne’s bioheat equation is analyzed, it can be concluded that in addition to the tumor size and location, electrical and thermal properties of the target tissue potentially influence the focusing as well as MH efficiency. It is expected that a late stage malignant tumor tissue will be large in size and an early stage malignant tumor will be small in size. Focusing can be more challenging for a small size tumor. On the other hand, the tumor can potentially be more homogeneous at an early stage, which can improve the effective energy deposition to the target tissues with distinctly different dielectric and thermal properties. The proposed method is patient-specific, and with the availability of stage-dependent tumor dielectric and thermal properties, different tumor stages can be evaluated. 

## 5. Conclusions

A straightforward microwave hyperthermia approach for the treatment of breast cancer was proposed. The simple nature of the linear antenna arrays and focusing maps enables the focusing of the heating potential to the target region without the need for complicated algorithms. With the presented method, 0.3 cm tumors, corresponding to early-stage tumors, can be focused even if they are embedded deep inside the dense tissue. 

Instead of giving the normalized values, it is worth mentioning that based on the structure of the breast, the value of HP varies significantly. In future, the total dissipated power needs to be carefully adjusted before further application. Furthermore, the Vivaldi antenna is an acceptable antenna for microwave imaging, and the proposed configuration enables the possibility of combining a hyperthermia applicator with the microwave imaging techniques [24,25,26].
